# Detecting Depression Using an Ensemble Logistic Regression Model Based on Multiple Speech Features

**DOI:** 10.1155/2018/6508319

**Published:** 2018-09-24

**Authors:** Haihua Jiang, Bin Hu, Zhenyu Liu, Gang Wang, Lan Zhang, Xiaoyu Li, Huanyu Kang

**Affiliations:** ^1^Faculty of Information Technology, Beijing University of Technology, Beijing 100124, China; ^2^Gansu Provincial Key Laboratory of Wearable Computing, School of Information Science and Engineering, Lanzhou University, Lanzhou 730000, China; ^3^Beijing Anding Hospital of Capital Medical University, Beijing 100088, China; ^4^Lanzhou University Second Hospital, Lanzhou 730030, China

## Abstract

Early intervention for depression is very important to ease the disease burden, but current diagnostic methods are still limited. This study investigated automatic depressed speech classification in a sample of 170 native Chinese subjects (85 healthy controls and 85 depressed patients). The classification performances of prosodic, spectral, and glottal speech features were analyzed in recognition of depression. We proposed an ensemble logistic regression model for detecting depression (ELRDD) in speech. The logistic regression, which was superior in recognition of depression, was selected as the base classifier. This ensemble model extracted many speech features from different aspects and ensured diversity of the base classifier. ELRDD provided better classification results than the other compared classifiers. A technique for identifying depression based on ELRDD, ELRDD-E, was here suggested and tested. It offered encouraging outcomes, revealing a high accuracy level of 75.00% for females and 81.82% for males, as well as an advantageous sensitivity/specificity ratio of 79.25%/70.59% for females and 78.13%/85.29% for males.

## 1. Introduction

Worldwide, over 300 million people of different ages have clinical depression [1]. The rise in the prevalence of this disease has been connected to a group of important outcomes [2]. At the most extreme, patients with depression may commit suicide [3]. To halt the onset of clinical depression, advance intervention can offer a pivotal action to ease the burden of the disease. However, current depression diagnosis methods rely on self-report of patient and clinical opinion [4], which risk several subjective biases. Therefore, a convenient and objective method for detecting depression is of primary importance.

Depressed speech is distinguished invariably by clinicians as monotone, uninteresting, and spiritless [5]. The acoustic qualities of speech can be affected by the emotional state of a person with depression [6]. Therefore, depression can be detected by analyzing changes in the acoustical characteristics of speech. Several approaches have been proposed to reveal correlations between depression and acoustic features for depressed speech classification. To improve the effect of classification, many features were extracted in early studies. However, it is still unclear which acoustic features are most effective for detecting depression especially in Mandarin speech. Furthermore, an objective method based on speech is still in need.

This study investigates the classification performance of multiple speech features which were extracted from subjects to identify depression in those who spoke Mandarin language. To develop an effective objective method and improve the classification result, we propose an ensemble logistic regression model for detecting depression (ELRDD), which contributes to depression recognition based on speech in several ways. First, to make the best use of speech features, it extracts many speech features from different aspects and ensures diversity of the feature spaces and the base classifiers. Second, to overcome the problem of dimensionality curse, the feature subspace dimensionality of each base classifier is lower than the all features space, while a feature reduction method is also used to avoid the curse of dimensionality. Third, a logistic regression model as the base classifier offers probabilities for every class, so the ensemble classifier could make the greatest use of the uncertain information to acquire the best classification outcomes.

The rest of the paper is structured as follows: Section 2 reviews the related work.  Section 3 describes the speech database used for this study.  Section 4 provides a detailed description of our methodology.  Section 5 describes the experiments and results, and Section 6 presents the conclusions.

## 2. Related Work

Darby and Hollien [7] performed an introductory evaluation of patients with major depression, and they discovered that listeners could discern various distinct characteristics in depressed speech. A variety of speech features have been explored for detecting depression. Mundt et al. [4], Stassen et al. [8], and Hönig et al. [9] reported correlations between F_0_ variables and depression. However, Alpert et al. [10], Cannizzaro et al. [11], and Yang et al. [12] reported no significant correlation between F_0_ variables and depression. Low et al. [13], Moore et al. [14], and Ooi et al. [15, 16] evaluated classification systems with prosodic, glottal, and spectral features. Low et al. [17], Valstar et al. [18], Alghowinem et al. [19], and Jiang et al. [20] used low-level descriptors and statistical characteristics to identify depression. Cummins et al. [21, 22], Sturim et al. [23], Alghowinem et al. [24], and Joshi et al. [25] investigated mel-frequency cepstrum coefficients (MFCC) and found that the recognition performance was statistically significant for depression classification. An evaluation by Scherer et al. [26–28] revealed a tight connection between voice quality features and the degree of depression. Quatieri and Malyska [29] and Ozdas et al. [30] discovered that depressed subjects showed increased energy levels on the glottal spectrum.

The support vector machine (SVM) and the Gaussian mixture model (GMM) are the most popular classification technologies used for detecting depression in speech. Moore et al. [14] studied 15 depressed subjects and 18 healthy controls and used quadratic discriminant analysis to construct a classifier. They reported accuracies of 91% (with sensitivity to specificity 89%/93%) for males and 96% (with sensitivity to specificity 98%/94%) for females. Their analysis showed that glottal features were more discriminating than prosodic features. Cohn et al. [31] recruited 57 depressed patients and used fundamental frequency and speak-switch duration as inputs to a logistic regression (LR) classifier. They reported an accuracy of 79% (with sensitivity to specificity 88%/64%) when classifying subjects who either responded or did not respond to treatment for depression. Low et al. [13] examined 139 adolescents (71 healthy and 68 depressed) who spoke English, and they used a gender-independent GMM classifier that incorporated glottal, prosodic, and spectral features. They reported classification results of 67–69% for males and 70–75% for females. Ooi et al. [16] studied 30 participants (15 were at risk of depression and 15 were not at risk) who spoke English and presented an ensemble method using GMM classifiers that used prosodic and glottal features. They reported a classification result of 74% (with sensitivity to specificity 77%/70%). Alghowinem et al. [32] recruited 30 controls and 30 depressed patients who spoke English. They summarized low-level descriptors and statistical features and compared the following classifiers: SVM, GMM, Multilayer Perceptron Neural Network (MLP), and Hierarchical Fuzzy Signature (HFS). They concluded that SVM and GMM had better classification performance. Helfer et al. [33] studied 35 subjects whose Hamilton Depression Scale (HAMD) scores were below 7 or above 17, respectively. They used associated dynamic and the first three formant trajectories as features and reported that SVM performed better than GMM when classifying depression severity. Jiang et al. [20] studied 170 subjects and proposed a computational methodology based on SVM (STEDD). They documented accuracies of 75.96% (with sensitivity to specificity of 77.36%/74.51%) for females and 80.30% (with sensitivity to specificity of 75.00%/85.29%) for males. It should be noted that most of these previous studies were usually limited to small depressed samples and focused on participants who spoke Western languages.

To the best of our knowledge, there has been little research exploring the ensemble classifier for detecting depression based on speech. However, ensemble logistic regression has been used effectively in other research fields [34–39]. In these previous studies, two methods were used to deal with the feature spaces. In one method, all feature spaces were used in each base classifier [34–36]. In the other method, the feature spaces were randomly partitioned into several subspaces [37–39]. It should be mentioned that the feature subspace dimensionality of the previous remained higher, and the variety of the feature subspaces of the last-mentioned could not be guaranteed and the classification outcome was unsteady.

## 3. Speech Database

In our research, all the subjects were native Chinese speakers between the ages of 18 and 55 and had at least an elementary school education [40]. First, every participant was required to fill in a preassessment booklet that contained general information and demographic information, including health history, age, gender, educational status, and employment. Second, every participant was chosen by psychiatrists based on the *Diagnostic and Statistical Manual Of Mental Disorders* (DSM-IV) [41] rules. Finally, all the subjects were interviewed by psychiatrists to complete the patient health questionnaire-9 (PHQ-9) [42]. These subjects were then divided into two groups depending upon the PHQ-9 scores: depressed patients (PHQ-9 ≥ 5) and healthy controls (PHQ-9 < 5). Depressed patients were diagnosed as having pure depression, and they did not experience any other mental illnesses. The controls had no previous or ongoing mental disorder and were matched to the depressed patients based on demographics.

Following the completion of the clinical evaluations, our recording experiment began and it consisted of three parts: an interview assignment, a reading assignment, and a picture detailing assignment. The interview assignment was made up of 18 questions, and the topics were taken from the Self-Rating Depression Scale (SDS), HAMD, and DSM-IV. The following are sample questions: How do you evaluate yourself? What is the most important present you have ever been given, and how did it make you feel? What do you enjoy doing when you are not able to fall asleep? Please detail any plans you may have for an upcoming vacation. Please tell us about a friend, including their age, type of employment, personality, and pastimes. What situations could make you become desperate? The reading assignment consisted of a short story named “*The North Wind and the Sun*” [43] and three sets of words with neutral (e.g., center, since), positive (e.g., outstanding, happy), and negative (e.g., depression, wail) emotions. The picture detailing assignment involved four dissimilar pictures. Three of them, which had neutral, positive, and negative faces, were obtained from the Chinese Facial Affective Picture System (CFAPS). The last picture titled “*Crying Woman*” was chosen from the Thematic Apperception Test (TAT) [9]. In this assignment, participants were requested to openly detail the four pictures.

We collected speech recordings in a quiet, soundproof, clean laboratory. The ambient noise level in the laboratory was kept below 60 dB. The speech signals were documented with a 24-bit sampling depth and 44.1 kHz sampling rate. We segmented and labeled all these recordings manually and retained only subject voice signals. These recordings were stored in an uncompressed WAV format. The database utilized in this evaluation contained speech recordings from 85 controls (34 males and 51 females) and 85 depressed individuals (32 males and 53 females). The speech of each subject was split into 29 recordings depending on different subtasks. In all, this study utilized 4,930 speech recordings. The overall lengths of speech during the interview, picture detailing, and reading were 52,427 s, 16,203 s, and 21,425 s, respectively. The average duration of speech recording was 18.3 s.

## 4. Methods

**Figure 1:**
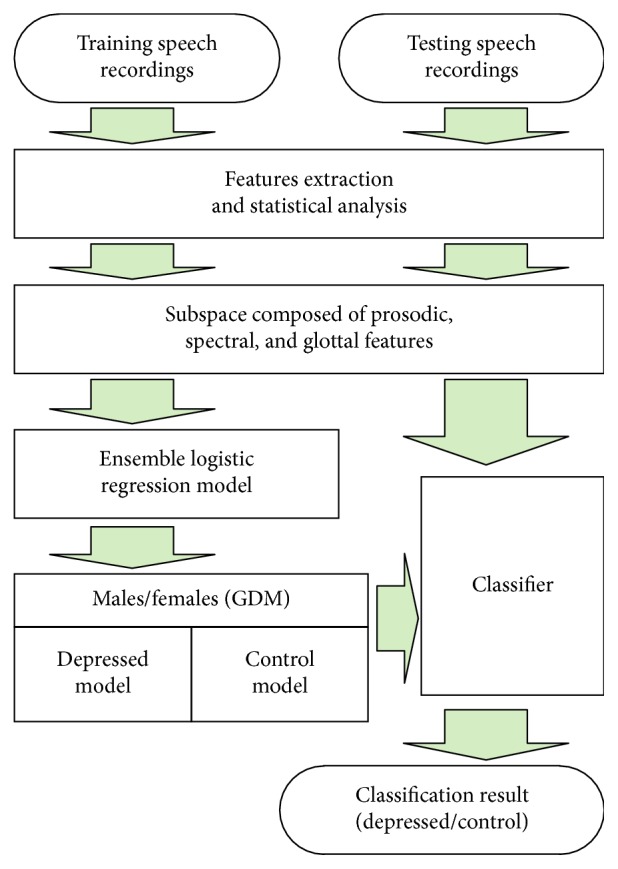
Block diagram of the ensemble logistic regression model for detecting depression (ELRDD).

In light of gender variations in depressive indications [44], there are two classification methods: gender-independent modeling (GIM) and gender-dependent modeling (GDM). Low et al. [13] discovered that GDM outperformed GIM. In our study, we used GDM, in which females and males were modeled independently. The proposed framework for the ELRDD is detailed in Figure 1. In the next sections of our paper, features extraction, features reduction, and modeling techniques are recounted.

### 4.1. Features Extraction and Reduction

The acoustic speech features explored in the literature can be divided into three main categories: prosodic features, spectral features, and glottal features. Each of the three categories comprises several subcategories. MFCC was one of the most frequent spectral features utilized in speech parameters, and the classification outcomes were statistically significant in identifying depression [22–25]. Therefore, MFCC was separated from spectral features as a main category. For convenience, the prosodic features are abbreviated PROS, the spectral features are abbreviated SPEC, and the glottal features are abbreviated GLOT.  Table 1 presents a summary of the main speech feature categories, subcategories, the number of features, and the statistical functions. Since PROS, SPEC, MFCC, and GLOT were extracted using very different feature extraction methods, they can describe speech from diverse aspects. Thus, feature vectors were complementary to one another. Then, if the feature subspaces of the ensemble classifier were made up of a few of these feature vectors, these subspaces will have a larger diversity. We combined one or more of these four features to form 15 different feature spaces.  Table 2 displays these subspaces made up of various feature vectors, in which PROS + SPEC suggests that the subspace is made up of the feature vectors of PROS and SPEC, and MFCC + PROS + SPEC + GLOT suggests that the space is made up of every one of the features. The glottal features were calculated using the TTK Aparat toolbox [45], and the prosodic and spectral features were calculated using the open-source software openSMILE [46].

Compared with the dimensionality of the whole feature space, the dimensionalities of feature subspaces had been reduced considerably, but some dimensionalities of the subspaces were still very high. We applied and compared principal component analysis (PCA), kernel PCA, Laplacian, Isomap, Landmark Isomap, and locally linear embedding (LLE) to reduce feature space dimensionality. We employed LLE, because it outperformed other methods and preserved the local geometry of high dimensional data [47].

### 4.2. Ensemble Classification

Given that training data *X*={*X*^(1)^, *X*^(2)^,…, *X*^(*K*)^} and its label *Y*={*Y*^(1)^, *Y*^(2)^,…, *Y*^(*K*)^}, where *X*^(*k*)^={*x*_1_^(*k*)^, *x*_2_^(*k*)^,…, *x*_*N*_^(*k*)^} is one of the feature subspaces of training points, the value of each label in *Y*^(*k*)^={*y*_1_^(*k*)^, *y*_2_^(*k*)^,…, *y*_*N*_^(*k*)^} was set to 1 for the depressed patients and 0 for the controls. Given test input data *x*={*x*^(1)^, *x*^(2)^,…, *x*^(*K*)^}, where *x*^(*k*)^ included in Table 2 is a feature subspaces of *x*, the outputs *P*(*L*=1|*x*) and *P*(*L*=0|*x*) providing the 1 and 0 estimated probabilities are given by

(1) $$\begin{array}{c} P\left(\left.L=1\right|x\right)=\overset{K}{\underset{k=1}{\sum }}P\left(\left.L=1\right|x^{\left(k\right)};w^{\left(k\right)}\right)=\overset{K}{\underset{k=1}{\sum }}\frac{\exp\left(w^{\left(k\right)}\cdot x^{\left(k\right)}\right)}{1+\exp\left(w^{\left(k\right)}\cdot x^{\left(k\right)}\right)}, \end{array}$$

(2) $$\begin{array}{c} P\left(\left.L=0\right|x\right)=\overset{K}{\underset{k=1}{\sum }}P\left(\left.L=0\right|x^{\left(k\right)};w^{\left(k\right)}\right)=\overset{K}{\underset{k=1}{\sum }}\frac{1}{1+\exp\left(w^{\left(k\right)}\cdot x^{\left(k\right)}\right)}, \end{array}$$

where *w*={*w*^(1)^, *w*^(2)^,…, *w*^(*K*)^} are the parameters of the ensemble logistic regression model. The log-likelihood function under this model is as follows:

(3) $$\begin{array}{c} l\left(w\right)=\overset{K}{\underset{k=1}{\sum }}l\left(w^{\left(k\right)}\right)=\overset{K}{\underset{k=1}{\sum }}\overset{N}{\underset{i=1}{\sum }}\left[y_{i}^{\left(k\right)}\left(w^{\left(k\right)}\cdot x_{i}^{\left(k\right)}\right)-\log\left(1+\exp\left(w^{\left(k\right)}\cdot x_{i}^{\left(k\right)}\right)\right)\right], \end{array}$$

where maximizing *l*(*w*) produces a maximum likelihood estimator for *w*.

According to Section 4.1, the complete algorithm of ELRDD is outlined in Algorithm 1.

To validate ELRDD, SVM, GMM, and LR were compared as classifiers for detecting depression. SVM and GMM were usually employed for recognition of depression, while LR was taken as the base classifier for ELRDD. We utilized the expectation-maximization (EM) algorithm to approximate the GMM parameters of every Gaussian component and a radial basis function (RBF) as SVM's kernel function. Then, we looked for the most adequate parameters with a grid search utilizing five-fold cross validation on our training dataset with the LIBSVM toolbox [48].

To demonstrate that ELRDD outperforms other ensemble classifiers, three classic classifiers were compared: adaboost decision tree, bagging decision tree, and random forest. They depicted the speech recordings by the feature spaces made up of MFCC, PROS, SPEC, and GLOT. Because males and females were modeled separately, the number of base classifiers for each classifier depended on gender. These numbers were chosen from 15, 50, 100, 200, 300, 400, and 500, which yielded the best classification outcomes.

ELRDD computed the probabilities that each speech recording belonged to depressed and healthy subjects. To improve recognition performance, classifying a subject as depressed patient or healthy control could use the classification results of more than one speech recording. In our study, each participant had 29 speech recordings, and the final classification result could depend on all these speech recordings. Therefore, we proposed ELRDD-E, which can be summarized as Algorithm 2.

We examined the precise classifications of the controls and the depressed patients in sensitivity, specificity, and accuracy. The controls were distinguished as the negative cases, and the depressed patients were distinguished as the positive cases. When examining performance, each of the three parameters of a well-performing method would have high values, but if a compromise was required, it was sensible to acquire the greatest accuracy while obtaining an optimum sensitivity/specificity ratio (ideally > 1). We employed a speaker-independent split of test and train data and used a ten-fold cross validation. The one-way analysis of variance (ANOVA) and the least significant difference (LSD) tests were conducted to establish if variations in the classification outcomes were statistically significant. The level of significance was set as *p* < 0.05.

## 5. Experiments and Results

### 5.1. Experiment Using Individual and Ensemble Classifiers for Males

Table 3 reveals the classification outcomes of every individual classifier for males. It can be noted that the chosen speech features impacted the recognition performance of classifiers. For example, SVM had the best specificity and accuracy with SPEC + GLOT. In contrast, LR achieved the best specificity and accuracy with PROS + SPEC, and GMM achieved the best accuracy with MFCC + PROS + SPEC. In addition, ANOVA and LSD tests were carried out on the four speech feature subspaces (MFCC, PROS, SPEC, and GLOT) over the ten-fold cross validation outcomes utilizing SVM, GMM, and LR classifiers. The accuracy, sensitivity, and specificity significantly varied between the four feature subspaces (*p* < 0.05). The accuracy and sensitivity of GLOT were worse in comparison to MFCC, PROS, and SPEC (*p* < 0.05), and the accuracy and specificity of SPEC and PROS were greater than MFCC (*p* < 0.05). ANOVA and LSD tests were also carried out on paired classifiers over the ten-fold cross validation outcomes. The specificity and accuracy of SVM, GMM, and LR were alike (*p* > 0.05), and the sensitivity of LR and GMM was greater than SVM (*p* < 0.05).

Table 4 shows the recognition performance of ELRDD and existing ensemble classifiers for males. The number of base classifiers for adaboost decision tree, bagging decision tree, and random forest was set to 300, 500, and 400, respectively, which yielded the best classification results. From Tables 3 and 4, it was discovered that ELRDD outperformed the greatest outcome of individual classifiers in accuracy and sensitivity in the identification of depression. Following ANOVA and LSD tests being conducted on paired ensemble classifiers over the ten-fold cross validation outcomes, we discovered that ELRDD also outperformed the contrasted current ensemble classifiers for males in sensitivity and accuracy (*p* < 0.05), and specificity was alike (*p* > 0.05).

### 5.2. Experiment Using Individual and Ensemble Classifiers for Females

Table 5 reveals the classification outcomes of every individual classifier for females. Following ANOVA and LSD tests being carried out on paired classifiers over the ten-fold cross validation outcomes, it can be noted that LR functioned as well as SVM and GMM (*p* > 0.05), and the greatest experimental outcome of LR outperformed SVM and GMM, which was in agreement with the outcomes for males. In addition, ANOVA and LSD tests were also conducted on the four speech feature subspaces (MFCC, PROS, SPEC, and GLOT) over the ten-fold cross validation outcomes utilizing SVM, GMM, and LR classifiers for females. The accuracy and specificity significantly varied between the four feature subspaces (*p* < 0.05). The accuracy and specificity of GLOT were worse than that of MFCC, PROS, and SPEC (*p* < 0.05), and the sensitivity of PROS was better than that of GLOT (*p* < 0.05).

Table 6 shows the recognition performances of ensemble classifiers for females. The number of base classifiers for adaboost decision tree, bagging decision tree, and random forest was set to 200, 300, and 300, respectively, which yielded the best classification results. After the LSD test, ELRDD still outperformed the other ensemble classifiers for females in terms of sensitivity (*p* < 0.05), and specificity and accuracy were similar (*p* > 0.05).

### 5.3. Experiment Using ELRDD-E

The classification outcomes of ELRDD-E are presented in Table 7. The outcomes of utilizing STEDD [20], which is an efficient technique according to speech types and emotions to identify depression in the identical database, are also included for comparison. From this table, it was found that ELRDD-E outperformed the results of Adaboost Decision Tree, Bagging Decision Tree, and Random Forest in terms of accuracy and sensitivity (*p* < 0.05). It also can be noted that ELRDD-E performed greater than STEDD in classification sensitivity and accuracy for males, while they had the same specificity. Further, ELRDD-E provided better sensitivity than STEDD for females, while STEDD performed minutely better in specificity and accuracy. It can be concluded that ELRDD-E provided very promising results and was effective for detecting depression.

## 6. Discussion

Table 3 shows the classification outcomes of each individual classifier for males. It can be noted that the optimal features for every classifier varied. These results indicate that each feature vector could provide complementary information for the different classifiers. Moreover, it was impossible for each classifier to utilize the same feature subspace that worked best for other classifiers. This indirectly indicates that it is necessary to develop classifiers from multiple feature subsets. Results showed that SPEC and PROS features performed better than MFCC and GLOT features for males.

Table 5 reveals the classification outcomes of every individual classifier for females. It can be observed that each classifier yielded the best classification result using different feature subspaces. These outcomes suggest that every feature was complementary and offered various classifiers with different information, which was also in agreement with the discoveries for males. It was noted that utilizing SPEC, PROS, and MFCC features offered significantly better classification outcomes for females compared to utilizing GLOT features.

From Tables 3 and 5, it can be concluded that using GLOT features provided worst classification outcomes among these four feature vectors. This result is contrary to the findings of two earlier studies. Low et al. [13] and Ooi et al. [15] observed that glottal features performed better than prosodic and spectral features. The disparity may be due to the fact that previous researchers focused on participants who spoke Western languages, while all the participants in this work spoke Mandarin. The assignments used in the previous studies were also different from ours. It also can be observed that the performance of LR was no worse than that of SVM and GMM with most feature subspaces. Furthermore, the best experimental result of LR outperformed SVM and GMM. This was one of the reasons that LR was chosen as the base classifier.

Tables 4 and 6 show the recognition performances of classifiers. It can be observed that ELRDD had a better recognition effect than other classifiers. This result could be due to the fact that ELRDD could ensure the diversity of the feature subspaces and utilize more information provided by features. Moreover, compared with the other three existing ensemble classifiers, the number of base classifiers in ELRDD was much smaller.

## 7. Conclusion

In this evaluation, we initially contrasted the outcomes of three varying individual classifiers utilizing 15 feature subspaces to determine the connection between speech features and the performance of classifiers. It was observed that classifier performance was sensitive to the features used for both males and females. Since each feature subspace contained different information of the speech recordings, it was reasonable to integrate suitable speech features. It was noted that utilizing SPEC and PROS features offered significantly better classification outcomes for males than utilizing MFCC and GLOT features (*p* < 0.05). It was discovered that utilizing GLOT features offered significantly worse classification outcomes for females than utilizing SPEC, PROS, and MFCC features (*p* < 0.05). It was also discovered that LR performed minutely better than SVM and GMM, which was a reason for LR being selected as the base classifier.

Second, we revealed an ensemble methodology for the classification of depression, ELRDD. It was noted that ELRDD, which was developed from multiple feature subsets, outperformed both the individual classifiers and the other ensemble classifiers including SVM, GMM, LR, adaboost decision tree, bagging decision tree, and random forest. ELRDD revealed an accuracy level of 70.64% for males and 66.68% for females, as well as a sensitivity/specificity ratio of 67.35%/73.94% for males and 65.71%/67.68% for females.

Finally, based on ELRDD, we proposed ELRDD-E, which utilized the classification results of all 29 speech recordings of each subject in our dataset. This methodology offered extremely encouraging outcomes, revealing an increased accuracy level of 81.82% for males and 75.00% for females, as well as an advantageous sensitivity/specificity ratio of 78.13%/85.29% for males and 79.25%/70.59% for females.

While the experimental outcomes are promising, a possible limitation of this research is that speech may have additional features that pertain to depression. A future direction of this study is to investigate improvements in feature extraction and selection strategy.

## Figures and Tables

**Algorithm 1:**
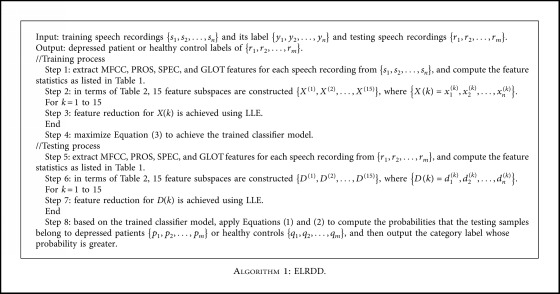
ELRDD.

**Algorithm 2:**
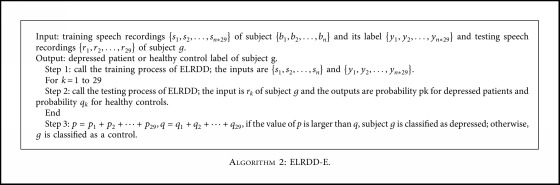
ELRDD-E.

**Table 1:** Summary of speech features.

Main category	Subcategory	Number of features	Functions
MFCC	MFCC (0–14)	630	Corresponding delta coefficients appended
SPEC	Flux	42	21 functions utilized
	Centroid	42	maxPos, minPos
	Entropy	42	Mean, std dev
	Roll-off	168	Skewness, kurtosis
	Band energies	84	Quartile 1/2/3
PROS	PCM loudness	42	Quartile range (2–1)/(3–2)/(3–1)
	Log mel-frequency band (0–7)	336	Linear regression error Q/A
	LSP frequency (0–7)	336	Linear regression coeff. 1/2
	F_0_ envelope	42	Percentile 1/99
	Voicing probability	42	Percentile range (99–1)
	F0final, ShimmerLocal	76	19 functions by eliminating the minimum value and the
	JitterLocal, JitterDDP	76	Range functions from the 21 abovementioned functions
	Pitch onsets, duration	2	No functions
GLOT	GLT	27	Mean, max, min
	GLF	5	Mean, max, min
Total		1992	

**Table 2:** Subspaces composed of several different feature vectors.

No.	Subspace	No.	Subspace	No.	Subspace
1	MFCC	2	PROS	3	SPEC
4	GLOT	5	MFCC + PROS	6	MFCC + SPEC
7	MFCC + GLOT	8	PROS + SPEC	9	PROS + GLOT
10	SPEC + GLOT	11	MFCC + PROS + SPEC	12	MFCC + PROS + GLOT
13	MFCC + SPEC + GLOT	14	PROS + SPEC + GLOT	15	MFCC + PROS + SPEC + GLOT

**Table 3:** Classification outcomes of each individual classifier for males.

Features	SVM			GMM			LR		
	Sen. (%)	Spe. (%)	Acc. (%)	Sen. (%)	Spe. (%)	Acc. (%)	Sen. (%)	Spe. (%)	Acc. (%)
MFCC	56.14	64.91	60.66	62.72	58.22	60.40	62.50	60.75	61.60
PROS	61.96	70.39	66.30	61.75	74.14	68.13	63.15	71.10	67.24
SPEC	**63.36**	73.94	68.81	65.84	71.60	68.81	**67.35**	70.69	69.07
GLOT	36.32	60.95	49.01	47.95	54.26	51.20	44.07	54.56	49.48
MFCC + PROS	60.67	69.78	65.36	63.69	70.49	67.19	65.41	68.36	66.93
MFCC + SPEC	59.05	72.72	66.09	64.55	69.17	66.93	63.58	69.07	66.41
MFCC + GLOT	53.56	66.53	60.24	61.96	60.65	61.29	61.10	60.75	60.92
PROS + SPEC	63.25	73.83	68.70	62.72	74.14	68.60	67.13	**72.21**	**69.85**
PROS + GLOT	60.99	71.60	66.46	61.96	72.92	67.61	62.82	71.20	67.14
SPEC + GLOT	62.61	**75.15**	**69.07**	65.19	70.89	68.13	66.70	70.99	68.91
MFCC + PROS + SPEC	60.99	72.92	67.14	65.63	72.31	**69.07**	64.55	70.39	67.56
MFCC + PROS + GLOT	59.59	72.62	66.30	63.36	69.27	66.41	62.82	67.24	65.10
MFCC + SPEC + GLOT	60.24	72.82	66.72	65.84	69.98	67.97	64.66	68.66	66.72
PROS + SPEC + GLOT	60.02	73.83	67.14	62.82	**74.24**	68.70	64.12	71.91	68.13
MFCC + PROS + SPEC + GLOT	61.85	73.12	67.66	**66.27**	71.30	68.86	64.33	**72.21**	68.39

Maximum of sensitivity (sen.), specificity (spe.), and accuracy (acc.) is shown in bold.

**Table 4:** Recognition performance of each classifier for males.

Classifier	Number of base classifiers	Sensitivity (%)	Specificity (%)	Accuracy (%)
Adaboost decision tree	300	58.94	67.14	63.17
Bagging decision tree	500	59.48	70.28	65.05
Random forest	400	59.05	70.99	65.20
ELRDD	15	67.35	73.94	70.64

**Table 5:** Classification outcomes of each individual classifier for females.

Features	SVM			GMM			LR		
	Sen. (%)	Spe. (%)	Acc. (%)	Sen. (%)	Spe. (%)	Acc. (%)	Sen. (%)	Spe. (%)	Acc. (%)
MFCC	63.24	57.27	60.31	56.47	66.06	61.17	62.79	61.80	62.30
PROS	67.21	60.65	63.99	51.72	73.29	62.30	64.35	66.73	65.52
SPEC	60.64	63.35	61.97	52.44	73.70	62.87	63.05	64.91	63.96
GLOT	56.60	42.53	49.70	51.33	50.44	50.90	52.70	46.11	49.47
MFCC + PROS	**67.53**	61.06	64.36	56.86	71.54	64.06	**64.93**	66.06	65.48
MFCC + SPEC	66.10	61.60	63.89	**57.78**	69.78	63.66	63.24	65.99	64.59
MFCC + GLOT	63.05	57.20	60.18	55.63	64.84	60.15	62.66	60.24	61.47
PROS + SPEC	64.09	**64.50**	64.29	51.01	**73.83**	62.20	63.63	**67.61**	65.58
PROS + GLOT	67.47	59.16	63.40	52.31	72.08	62.00	63.37	66.73	65.02
SPEC + GLOT	61.09	60.31	60.71	51.79	70.99	61.21	61.87	62.75	62.30
MFCC + PROS + SPEC	64.74	62.41	63.59	56.47	73.09	**64.62**	64.41	67.41	65.88
MFCC + PROS + GLOT	67.08	62.27	**64.72**	55.50	72.62	63.89	64.74	67.14	**65.92**
MFCC + SPEC + GLOT	63.37	62.68	63.03	57.71	69.37	63.43	62.39	63.42	62.90
PROS + SPEC + GLOT	64.15	63.42	63.79	51.53	73.43	62.27	63.11	67.07	65.05
MFCC + PROS + SPEC + GLOT	65.00	63.22	64.13	56.02	72.96	64.32	63.44	**67.61**	65.48

Maximum of sensitivity (sen.), specificity (spe.), and accuracy (acc.) are shown in bold.

**Table 6:** Recognition performance of each classifier for females.

Classifier	Number of base classifiers	Sensitivity (%)	Specificity (%)	Accuracy (%)
Adaboost decision tree	200	59.34	69.91	64.52
Bagging decision tree	300	58.75	68.56	63.56
Random forest	300	59.66	68.56	64.03
ELRDD	15	65.71	67.68	66.68

**Table 7:** Classification outcomes of ELRDD-E.

Gender	Classifier	Sensitivity (%)	Specificity (%)	Accuracy (%)
Male	ELRDD-E	78.13	85.29	81.82
	Adaboost decision tree	65.63	82.35	74.24
	Bagging decision tree	65.63	79.41	72.73
	Random forest	62.50	79.41	71.21
	STEDD	75.00	85.29	80.30
Female	ELRDD-E	79.25	70.59	75.00
	Adaboost decision tree	64.15	76.47	70.19
	Bagging decision tree	62.26	74.51	68.27
	Random forest	66.04	76.47	71.15
	STEDD	77.36	74.51	75.96

## Data Availability

The data used to support the findings of this study are available from the corresponding author upon request.
